# Cognitive Mechanisms Underlying the Influence of Facial Information Processing on Estimation Performance

**DOI:** 10.3390/bs15020212

**Published:** 2025-02-14

**Authors:** Xinqi Huang, Xiaofan Zhou, Mingyi Xu, Zhihao Liu, Yilin Ma, Chuanlin Zhu, Dongquan Kou

**Affiliations:** School of Educational Science, Yangzhou University, Yangzhou 225002, China; 210502108@stu.yzu.edu.cn (X.H.); 210502131@stu.yzu.edu.cn (X.Z.); 210502121@stu.yzu.edu.cn (M.X.); 210502112@stu.yzu.edu.cn (Z.L.); 210502113@stu.yzu.edu.cn (Y.M.)

**Keywords:** emotion priming, facial expression, estimation problems, math anxiety

## Abstract

This study aimed to investigate the roles of facial information processing and math anxiety in estimation performance. Across three experiments, participants completed a two-digit multiplication estimation task under the conditions of emotion judgment (Experiment 1), identity judgment (Experiment 2), and combined emotion and identity judgment (Experiment 3). In the estimation task, participants used either the down-up or up-down problem to select approximate answers. In Experiment 1, we found that negative emotions impair estimation performance, while positive and consistent emotions have a facilitating effect on estimation efficiency. In Experiment 2, we found that emotion and identity consistency interact with each other, and negative emotions actually promote estimation efficiency when identity is consistent. In Experiment 3, we found that emotion, identity consistency, and emotional consistency have complex interactions on estimation performance. Moreover, in most face-processing conditions, participants’ estimation performance is not affected by their level of math anxiety. However, in a small number of cases, mean proportions under happy and fearful conditions are negatively correlated with math anxiety.

## 1. Introduction

Estimation refers to the process in which individuals, without performing precise calculations, use certain rules or procedures to mentally approximate an arithmetic problem’s answer as closely as possible (Lemaire & Brun, 2017; Hinault & Lemaire, 2017). This process is particularly valuable for solving everyday problems. In psychological research, multiplication estimation problems have garnered significant attention. Commonly used problems include the rounding-down (RD) problem, where both factors disregard the units digit and retain only the tenth digits for multiplication (e.g., estimating 51 × 72 by considering it as 50 × 70). The rounding-up (RU) problem entails adjusting both numbers to the closest multiple of ten (e.g., estimating 49 × 67 as 50 × 70). The up-down (UD) problem involves increasing the first number to the nearest ten while retaining the second number’s ten as is (e.g., estimating 37 × 83 by considering it as 40 × 80). Finally, the down-up (DU) problem preserves the tenth digit of the initial number and adjusts the second number to the next highest multiple of ten (e.g., estimating 43 × 58 by considering it as 40 × 60) (Hinault & Lemaire, 2017; Zhu et al., 2021a).

Current research shows that estimation performance can be influenced by multiple factors, such as gender, emotional experience (Zhu et al., 2022a; Hill et al., 2016), age (Lemaire & Lecacheur, 2011; Wylie et al., 2012; Ai et al., 2017; Geurten & Lemaire, 2017), cognitive load (Imbo & Vandierendonck, 2007; Peng et al., 2016), and cognitive style (Yan, 2010). These factors jointly affect individuals’ cognitive resources, influencing the stability and adaptability of estimation problems in different contexts. Mathematics anxiety, defined as an individual’s emotional and cognitive fear of mathematics, has been confirmed by multiple scholars to impact mathematical performance (Ganley & Vasilyeva, 2011; Ramirez & Beilock, 2011; Živković et al., 2022; Lau et al., 2024). It is noteworthy that the mathematics anxiety effect (Hopko et al., 1998)—where individuals with mathematics anxiety experience intrusive thoughts and worries during math-related tasks and have difficulty inhibiting task-irrelevant information—depends on the task’s difficulty. For example, Ashcraft and Faust (1994) noted an anxiety–complexity effect: for some low-difficulty tasks, there is no performance difference between individuals with high and low mathematics anxiety, but as tasks become more complex or difficult, the effect of mathematics anxiety becomes more pronounced. The high-anxiety group had the lowest mean proportions in mental arithmetic tasks, while the low-anxiety group was faster than the moderate-anxiety group in task completion. Some studies also revealed that participants experienced increased levels of mathematics anxiety when engaged in complex mathematical tasks as opposed to simpler ones (Maki et al., 2024). Overall, behavioral studies suggest that the effect of mathematics anxiety is task-dependent; it is unlikely to manifest in simple tasks but becomes evident when task difficulty reaches a certain threshold.

Emotion is a significant factor influencing estimation problems. In addition to research on anxiety, numerous studies on other emotions have confirmed this. To investigate this issue, Fabre and Lemaire selected positive, neutral, and negative images from the International Affective Picture System as emotional stimuli to induce participants’ emotional experiences of different valences and then had them complete a multiplication arithmetic verification task. The results showed that, compared to neutral emotional priming, negative emotional priming reduced task difficulty, while positive emotional priming increased task difficulty (Fabre & Lemaire, 2019). This study was the first to examine the impact of induced emotional experiences on individuals’ performance in mathematical tasks under laboratory conditions, further deepening our understanding of the relationship between emotional experiences and mathematical performance. Subsequent research confirmed that negative emotional experiences not only weakened individuals’ estimation performance but also that this adverse effect was more pronounced in the young adult participant group (Lallement & Lemaire, 2021). Moreover, as an important component of facial information, emotion is crucial for decision-making and interpersonal communication. In addition to emotions, identity is also a key element in identifying facial information. Firstly, in the process of facial information processing, identity information is very important and is the most basic information, while emotional information is conveyed through expressions and contains rich social meanings. Therefore, the important status of both in the process of facial information processing is complementary. The processing of these types of facial information is not independent but occurs in parallel (Li & Tse, 2016; Lipp et al., 2014), indicating that different cognitive systems lead to varying processing outcomes.

In reviewing previous studies, regarding the relationship between math anxiety and estimation problems, Si et al. (2014) found that the level of math anxiety has little substantial impact on the mean proportions or response times of estimation problems. Similar findings related to the anxiety–complexity effect were confirmed in multiple studies (Doz et al., 2023; Maki et al., 2024). Moreover, the research by Maloney et al. (2010) suggested that working memory plays a moderating role in this context. Specifically, in purely numerical contexts, when solving simple multiplication estimation problems, participants may rely more on retrieval problems to quickly and directly extract factual knowledge from long-term memory. In contrast, when solving complex multiplication estimation problems, additional processes such as carrying numbers and tracking numerical values are required, meaning that both declarative and procedural knowledge must be engaged. In this sense, the mechanisms underlying the solution of simple and complex estimation problems are different, and math anxiety exerts distinct influences on them. It can be inferred that, during estimation processes, the impact of math anxiety on declarative knowledge is not significant. This ambiguity contrasts with the clear influence of emotional factors on estimation problems, the execution of which is modulated by the emotional value of emotional stimuli and the method of emotional priming. For example, when individuals use specific strategies to complete estimation tasks, negative emotional priming tends to impair estimation performance, while positive emotional experiences have a facilitating effect (Fabre & Lemaire, 2019; Liu et al., 2021). Additionally, research has shown that explicit fear priming can increase response times, while implicit fear priming helps to reduce response times. Both explicit and implicit happiness priming can enhance estimation performance (Zhu et al., 2021a; Liu et al., 2021). Stenberg et al. (1998) first proposed the emotional word-face Stroop task. In this experimental paradigm, emotional words are superimposed on faces with emotional valence, and participants are required to judge the emotional valence of the faces while ignoring the interference from the emotional words on the faces and to make a button-press response. The semantic meaning of the emotional word may be consistent with the facial expression (e.g., the word “happy” on a happy face) or inconsistent (e.g., the word “sad” on a happy face). The study found that response times for incongruent emotional stimuli are longer than those for congruent emotional stimuli, indicating the presence of an emotional congruence effect. Regarding the processing of facial emotional and identity information, studies have shown that they are not processed independently but occur in parallel (Li & Tse, 2016; Lipp et al., 2014), indicating that different cognitive systems can lead to different processing outcomes. For example, research has shown that different spatial frequency information can modulate the interaction between identity and emotional information (Wang et al., 2011), and facial identity recognition can affect the neural processing of emotional expressions, as evidenced by its impact on the N170 component during emotional recognition (Komatsu et al., 2020). Furthermore, based on the selective attention paradigm proposed by Ganel and Goshen-Gottstein (2004) (significant facial emotions do not affect facial identity judgments, but facial identity affects facial emotion judgments), they asked participants to judge the identity or emotion of faces, while the irrelevant dimension (i.e., the variable unrelated to the research objective) of identity or emotion remained unchanged (control condition) or changed (orthogonal condition). They found that response times for identity judgments were not affected by changes in emotion, while response times for emotion judgments were affected by changes in identity. Given this, the present study aims to explore the interaction between facial emotional and identity information processing and the selection of estimation answers. By examining the interaction of these cognitive systems in estimation performance, we aim to reveal the underlying cognitive mechanisms that control these processes, potentially providing a more nuanced understanding of the cognitive dynamics at play in social and mathematical contexts.

In this study, we set out to investigate the impact of processing facial emotional and identity information on the deployment of estimation problems and to unravel the cognitive mechanisms underlying these processes. To address these questions, a series of experiments were designed. In Experiment 1, participants were asked to first judge whether the two facial expressions presented on the left and right sides of the screen conveyed the same emotion (emotion judgment task) and then complete a two-digit multiplication estimation task using either the DU or UD problem (e.g., 21 × 38). Experiment 2 was similar to Experiment 1, except that the emotion judgment task was replaced by an identity judgment task (judging whether the two facial expressions presented were from the same person). Experiment 3 was similar to Experiment 1 but involved a combined emotion-identity judgment task (judging whether the two facial expressions presented were from the same person expressing the same emotion). In Experiments 1 and 3, which both involved emotions, there were two conditions: consistent and inconsistent. For the consistent condition, there were three scenarios: happy–happy, neutral–neutral, and fearful–fearful. For the inconsistent condition, there were three scenarios: happy–neutral, happy–fearful, and neutral–fearful. Our research is structured around three hypotheses designed to probe different aspects of the interaction between emotional and identity information and estimation problems.

**Hypothesis** **1.** *We posit that negative emotions will impair estimation performance, while positive emotional experiences will have a facilitating effect (Fabre & Lemaire, 2019; Liu et al., 2021). We anticipate that when facial expressions show happy–happy or happy–neutral, it will promote the estimation process, leading to quicker and potentially more accurate responses; on the other hand, when facial expressions show fearful–fearful or neutral–fearful, it will reduce estimation efficiency.*

**Hypothesis** **2.** *Based on the emotional consistency effect (Stenberg et al., 1998), we hypothesize that response times will be shorter under emotional consistent (vs. emotional inconsistent) conditions. In other words, emotional consistency is expected to enhance individuals’ estimation efficiency.*

**Hypothesis** **3.** *This hypothesis explores the interaction between facial emotional and identity information. Drawing from research on selective attention, we expect that facial emotion will not affect the judgment of facial identity, but the congruence of facial identity will significantly affect the response times in emotional judgment tasks (Ganel & Goshen-Gottstein, 2004).*

**Hypothesis** **4.** *This hypothesis addresses the relationship between mathematics anxiety and estimation performance. Previous research indicates that mathematics anxiety has no significant impact on the mean proportions and response times of estimation problem (Si et al., 2014). Based on this, we hypothesize that estimation performance, measured by mean proportions and response times, will not be significantly correlated with levels of mathematics anxiety across different conditions of facial information processing.*

## 2. Experiment 1: The Influence of Facial Emotion Information Processing on Estimation Performance

### 2.1. Methods

#### 2.1.1. Participants

The power analysis conducted using MorePower 6.0.4 indicates that, for a 2 (emotional consistency) × 2 (estimation problem types) × 3 (emotion types) experimental design, a minimum of 24 participants is required to ensure an effect size of $\eta_{p}^{2}$ = 0.26 (α = 0.05, power level = 0.95). When investigating the interaction between the type of emotional consistency (emotionally consistent and emotionally inconsistent), the type of emotion (happy, neutral, and fearful) and the type of estimation problem (UD and DU), 49 college students were recruited, forming a robust sample for this study. The study comprised individuals aged 18 to 24 years (M ± SD, 19.98 ± 1.38). All participants were exclusively right-handed and possessed either unimpaired vision or vision that had been adjusted to meet normal standards. This study was conducted according to the ethical principles of the Declaration of Helsinki and received approval from the Research Ethics Committee of the School of Education Science of Yangzhou University (JKY-2023032701). Following a detailed explanation of the study’s procedures, written consent was obtained from each participant. In return for their involvement, participants were provided with a small amount of money.

#### 2.1.2. Materials

(1) Facial expression images

The facial expression images used in this study were sourced exclusively from the NimStim Facial Expression Set (Tottenham et al., 2009). For the formal experiment, 54 facial expression images, featuring 18 distinct models, including 9 females, the database contained the following model identifiers: males—20, 21, 24, 27, 33, 36, 37, 41, and 42; females—1, 2, 3, 6, 9, 12, 13, 17, and 19. The images that had been previously validated were chosen, representing emotions of happiness, neutral, and fear. Twenty-five participants (not taking part in the formal experiment) were asked to assess the valence and arousal of all facial expression images by using a 9-point scale. When evaluating the valence of all facial expression images, “1” means very unpleasant, and “9” means very pleasant; when judging the arousal of all facial expression images, “1” stands for not at all arousing, and “9” stands for very arousing. The emotional combinations of the two faces include: happy–happy, neutral–neutral, fearful–fearful, happy–neutral, happy–fearful, neutral–fearful, neutral–happy, fear–happy, and fear–neutral, meaning there were a total of nine types. The number of emotional faces and neutral faces is equal. Each type has 24 groups. The emotional combinations of the first and second faces were random, and the number of combinations was completely consistent. The results of the repeated-measure ANOVA revealed a significant main effect of valence, *F*(2, 34) = 28.95, *p* < 0.001, $\eta_{p}^{2}$ = 0.630. Happiness (M ± SD, 6.23 ± 1.12) exhibited a greater valence compared to the valence associated with neutral (4.61 ± 0.75, *p* = 0.005) and fear (3.49 ± 0.84, *p* < 0.001), while the valence of neutral was higher than that of fear (*p* < 0.001). Concurrently, there was no significant primary effect observed for the dimension of arousal, *F*(2, 34) = 0.49, *p* = 0.617, and $\eta_{p}^{2}$ = 0.028. The arousal levels for the three emotions were as follows: happiness, M ± SD, 5.62 ± 0.49; neutral, 5.57 ± 0.50; fear, 5.70 ± 0.45.

In summary, these facial expression images exhibited significant differences in valence among different emotions under non-arousing conditions, thus allowing for an effective examination of the impact of rapid emotional congruence in valence—instead of arousal—on the implementation of estimation problems. The boundaries of each photograph were adjusted to an oval configuration employing Photoshop 8.0. The viewing angle was 4.52° (horizontal) × 6.75° (vertical), with a screen resolution of 100 pixels per inch.

(2) Multiplication formulas

A total of 216 two-digit multiplication problems were used, with 108 suitable for the DU problem and 108 for the UD problem. The problems were selected based on specific criteria from prior studies (Zhu et al., 2021b, 2022a, 2022b, 2023, 2024) to ensure optimal visibility and relevance to the problems, with the following details: (1) No operands had the closest decimal number equal to 10 (e.g., 12 × 69) or 100 (e.g., 23 × 98). (2) No operands had 0 (e.g., 21 × 60) or 5 as the unit digit (e.g., 45 × 57). (3) No operands had repeating digits in the tens (e.g., 21 × 27) or unit place (e.g., 67 × 87). (4) No digits were repeated within the operands (e.g., 22 × 37). (5) No tie problems were used (e.g., 38 × 38). The selection of the incorrect estimate was based on the following two specific criteria: (1) Method for calculating the incorrect estimate: For UD problems, the incorrect estimate was calculated by adding or subtracting 100 or 200 from the correct estimate to ensure it was plausibly incorrect but closely related to the correct answer. For example, if the correct estimate was 1900, the incorrect estimate might be 1800 or 2000. Additionally, for UD problems, the incorrect estimate was closer to the correct estimate than the estimate obtained using the DU strategy. The same approach was applied for calculating incorrect estimates for DU problems. (2) Position of the incorrect estimate: The correct and incorrect estimates had an equal probability of appearing on the left or right side to eliminate any potential bias in the results due to the position of the correct estimate. The multiplication problems were displayed on a black background in Times New Roman font, size 35.

(3) Mathematics anxiety rating scale

The Revised Mathematics Anxiety Rating Scale (R-MARS) (Si et al., 2011, 2014), consisting of 21 items, was used to assess participants’ mathematics anxiety. Participants rated their anxiety on a 5-point Likert-type scale, with “1” indicating “not anxious” and “5” indicating “very anxious”. For example, one item on the R-MARS might be “I feel anxious when I have to use the tables at the back of the math textbook”, to which participants would respond according to their level of anxiety experienced.

#### 2.1.3. Procedure

**Figure 1:**
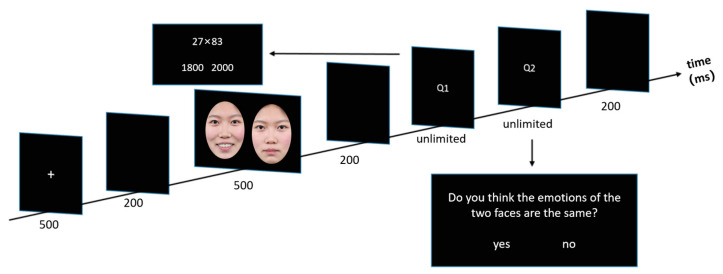
Trial structure in Experiment 1. The facial expression images used in the actual experiment were sourced from the NimStim Facial Expression Set, but due to copyright restrictions, the images in the figure are provided by the authors (Experiment 1).

This study took place within a small, acoustically insulated chamber. The experiment was administered using E-prime 2.0 (Psychology Software Tools, Inc., Pittsburgh, PA, USA). The experimental design was structured into four blocks, each consisting of 54 trials, resulting in 216 trials in total. As shown in Figure 1, the sequence of events for a single trial was detailed as follows: a white “+” symbol was displayed initially for 500 ms, succeeded by a 200 ms blank screen, then a facial expression image shown for 500 ms, after which followed another 200 ms blank screen, a multiplication computational estimation task (i.e., MCE task, Q1, with no time limit), an emotional consistency judgment task (i.e., ECJ task, Q2, with no time limit), and concluded with a final 200 ms blank screen. Before the commencement of the experiment, participants were provided with instructions on employing the DU and UD answers. They were tasked with using either the DU or UD problem to estimate the solution to each multiplication problem, with a focus on achieving precision. In the MCE task, the multiplication question was displayed in the middle of the screen, accompanied by two preselected answer choices symmetrically arranged below it. Participants were directed to respond by pressing the “F” key for answers they deemed to be on the left side, and the “J” key for those they perceived as being on the right side, corresponding to the position of the correct answer. After making their selection, the system then advanced automatically to the subsequent display. In the ECJ task, participants had to judge whether the pair of facial expression images expressed the same emotion, using the “F” key to indicate matching emotions and the “J” key for non-matching emotions. Participants were urged to balance mean proportions with speed in their responses during both the MCE and ECJ tasks.

This study adopted a pseudorandom design, with each experimental condition consecutively occurring no more than three times. In order to guarantee that participants grasped the research protocol, a set of 24 practice trials was offered prior to the commencement of the formal experiment. To mitigate the effects of fatigue, all participants were instructed to take a 1 min rest after finishing each set of tasks before moving on to the subsequent set. In the practice phase, participants received feedback, which was not offered during the formal experimental phase.

#### 2.1.4. Data Analysis

Experiment 1 adopted a 2 (emotional consistency) × 2 (estimation problem types) × 3 (emotion types) experimental design. Participants were tasked with performing an estimation task followed by an emotion consistency judgment task (judging whether the facial expressions conveyed the same type of emotion). The independent variables are the types of emotional information expressed by the two faces and the types of estimation question. Mean proportions and response times of participants in completing the MCE task were dependent variables. Inclusion in the analysis was limited to trials where subjects accurately finished the ECJ task. Experimental data were analyzed using ANOVA. Response times were analyzed for correctly solved problems. For trials with correct answers, participants’ response times were measured from the presentation of the multiplication formula to the pressing of the correct key. The analysis of data was performed with SPSS 26.0. A Pearson correlation coefficient was employed to assess the correlation between performance levels and mathematical anxiety. When the degrees of freedom did not meet the sphericity assumption, the Greenhouse–Geisser adjustment was implemented for *p*-values. All post hoc tests included Bonferroni corrections. The effect size was quantified using partial eta squared ($\eta_{p}^{2}$).

### 2.2. Results

#### 2.2.1. Mean Proportions and Response Times When Completing the MCE Task

Estimate selection was considered as correct if participants selected UD estimates for UD problems (e.g., a participant selected 1800 for 67 × 32) or if participants selected DU estimate for DU problems (e.g., a participant selected 2400 for 62 × 37). Otherwise, it was considered an incorrect response. We analyzed mean proportions and response times using a repeated-measure ANOVA. The results are detailed below.

For mean proportions, the interaction effect between emotion and emotional consistency was significant, *F*(1, 48) = 11.256, *p* = 0.002, $\eta_{p}^{2}$ = 0.190. The simple effect analysis showed that under emotional consistency conditions, mean proportions were higher in the happy condition than in the neutral condition (*p* < 0.001), higher in the happy condition than in the fear condition (*p* < 0.001), and higher in the neutral condition than in the fear condition (*p* = 0.003). Under emotional inconsistency conditions, mean proportions were higher in the happy condition than in the neutral condition (*p* < 0.001), higher in the happy condition than in the fear condition (*p* < 0.001), and lower in the neutral condition than in the fear condition (*p* = 0.045). In the neutral condition, mean proportions were higher under emotional consistency than under emotional inconsistency (*p* = 0.020). The main effect of emotion was significant, *F*(1, 48) = 194.169, *p* < 0.001, $\eta_{p}^{2}$ = 0.802. The main effect of emotional consistency was not significant, *F*(1, 48) = 2.037, *p* = 0.160, $\eta_{p}^{2}$ = 0.041. The main effect of estimation problem was significant, *F*(1, 48) = 28.268, *p* < 0.001, $\eta_{p}^{2}$ = 0.371. Post hoc analysis for estimation problems showed that mean proportions were higher for DU problems than for UD problems (*p* < 0.001). The interaction effect between emotion and estimation problem was not significant, *F*(1, 48) = 0.924, *p* = 0.341, $\eta_{p}^{2}$ = 0.019. The interaction effect between emotional consistency and estimation problem was not significant, *F*(1, 48) = 2.188, *p* = 0.146, $\eta_{p}^{2}$ = 0.044. The three-way interaction effect was not significant, *F*(1, 48) = 0.001, *p* = 0.982, $\eta_{p}^{2}$ = 0.000. The specific numerical values of mean proportions for completing estimation tasks under different conditions are shown in Table 1.

For response times, the interaction effect between emotion and emotional consistency was significant, *F*(1, 48) = 16.080, *p* < 0.001, $\eta_{p}^{2}$ = 0.251. The simple effect analysis showed that under emotional consistency conditions, response times were shorter in the happy condition than in the neutral condition (*p* = 0.014), shorter in the happy condition than in the fear condition (*p* < 0.001), and longer in the fear condition than in the neutral condition (*p* = 0.011). Under emotional inconsistency conditions, response times were shorter in the happy condition than in the neutral condition (*p* = 0.003) and shorter in the happy condition than in the fear condition (*p* = 0.023). In the happy condition, response times were shorter under emotional consistency than under emotional inconsistency (*p* < 0.001). In the neutral condition, response times were shorter under emotional consistency than under emotional inconsistency (*p* < 0.001). In the fear condition, response times were shorter under emotional consistency than under emotional inconsistency (*p* = 0.037). The main effect of emotion was significant, *F*(1, 48) = 44.528, *p* < 0.001, $\eta_{p}^{2}$ = 0.481. The main effect of emotional consistency was significant, *F*(1, 48) = 63.851, *p* < 0.001, $\eta_{p}^{2}$ = 0.571. The main effect of estimation problem was not significant, *F*(1, 48) = 0.139, *p* = 0.711, $\eta_{p}^{2}$ = 0.003. The interaction effect between emotion and estimation problem was not significant, *F*(1, 48) = 0.073, *p* = 0.788, $\eta_{p}^{2}$ = 0.002. The interaction effect between emotional consistency and estimation problem was not significant, *F*(1, 48) = 2.565, *p* = 0.116, $\eta_{p}^{2}$ = 0.051. The three-way interaction effect was not significant, *F*(1, 48) = 0.563, *p* = 0.457, $\eta_{p}^{2}$ = 0.012. The specific numerical values of response times for completing estimation tasks under different conditions is shown in Table 1. Additionally, the Pearson correlation analysis revealed no significant correlation between mean proportions and response times under different conditions (*ps* > 0.05) (Table 1), indicating that this study found no trade-off between response times and mean proportions.

#### 2.2.2. The Moderating Role of Math Anxiety in the Influence of Emotions on Estimation Performance

The results of Pearson correlation analysis indicated partial correlations between mean estimation proportions under different emotional conditions and math anxiety. Specifically, when participants faced emotional inconsistent faces under the happy condition, there was a negative correlation between mean estimation proportions for UD problems and math anxiety (*p* = 0.010), as well as between mean estimation proportions for DU problems and math anxiety (*p* = 0.009). When participants faced emotional inconsistent faces under the fear condition, there was a negative correlation between mean estimation proportions for UD problems and math anxiety (*p* = 0.024). No significant correlations were found between estimation response times under different emotional conditions and math anxiety (Table 2).

In summary, Experiment 1 demonstrated that, first, individuals’ mean estimation proportions and response times were more readily influenced by emotions and emotional consistency. Second, under different emotional conditions, there was a partial correlation between individuals’ performance in estimation tasks and their level of math anxiety, which was mainly manifested as a negative correlation between mean proportions and math anxiety under happy and fearful conditions when emotional inconsistency was present. Based on the known influence of facial emotion consistency on estimation problems, we further explored whether facial identity consistency would also affect the selection and execution of estimation answers. To this end, we designed relevant experiments for verification.

## 3. Experiment 2: The Influence of Facial Identity Information Processing on Estimation Performance

### 3.1. Methods

#### 3.1.1. Participants

The power analysis conducted using MorePower 6.0.4 indicates that, for a 2 (identity consistency) × 2 (estimation problem types) × 3 (emotion types) experimental design, a minimum of 24 participants is required to ensure an effect size of $\eta_{p}^{2}$ = 0.26 (α = 0.05, power level = 0.95). This study comprised 48 college students aged 18 to 24 years (M ± SD, 20.06 ± 1.33). All participants were exclusively right-handed and possessed either unimpaired vision or vision that had been adjusted to meet normal standards. This study was conducted according to the ethical principles of the Declaration of Helsinki and received approval from the Research Ethics Committee of the School of Education Science of Yangzhou University (JKY-2023032701). Following a detailed explanation of the study’s procedures, written consent was obtained from each participant. In return for their involvement, participants were provided with a small amount of money.

#### 3.1.2. Materials

The materials used in Experiment 2 were identical to those used in Experiment 1. The materials used in Experiment 2 were identical to those used in Experiment 1. However, the emotion of two faces was the same, meaning only consistent emotions were presented (happy–happy, neutral–neutral, and fearful–fearful). These were divided into two categories based on identity consistency (consistent identity and inconsistent identity), meaning there were a total of 6 types, with 36 groups of each type.

#### 3.1.3. Procedure

**Figure 2:**
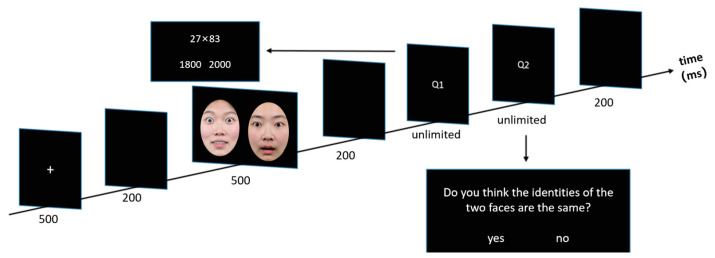
Trial structure in Experiment 2. The facial expression images used in the actual experiment were sourced from the NimStim Facial Expression Set, but due to copyright restrictions, the images in the figure are provided by the authors (Experiment 2).

The experimental procedure is the same as that of Experiment 1, but the emotional consistency judgment task in Experiment 1 has been supplanted by the identity consistency judgment (ICJ) task. Specifically, participants were instructed to press the “F” key if they perceived the two faces as having consistent identities, indicating that they belonged to the same individual, and to press the “J” key if they perceived the two faces as having inconsistent identities, suggesting that they belonged to different individuals. Figure 2 illustrates the order in which stimuli were presented during an individual trial, providing a clear visual representation of the sequence and timing of the stimuli presentation in the ICJ task.

#### 3.1.4. Data Analysis

The data analysis method used in Experiment 2 was the same as that used in Experiment 1.

### 3.2. Results

#### 3.2.1. Mean Proportions and Response Times When Completing the MCE Task

Following the pattern established in Experiment 1, we analyzed mean proportions and response times using repeated-measure ANOVA. The results are detailed below.

For mean proportions, the interaction effect between estimation problem and identity consistency was significant, *F*(1, 47) = 5.579, *p* = 0.022, and $\eta_{p}^{2}$ = 0.106. Simple effects analysis showed that, under identity consistency conditions, mean proportions for UD problems were lower than that for DU problems (*p* = 0.003). The main effect of emotion type was not significant, *F*(1, 47) = 0.952, *p* = 0.334, and $\eta_{p}^{2}$ = 0.020. The main effect of identity consistency was not significant, *F*(1, 47) = 0.930, *p* = 0.340, and $\eta_{p}^{2}$ = 0.019. The main effect of estimation problem was significant, *F*(1, 47) = 8.487, *p* = 0.005, and $\eta_{p}^{2}$ = 0.153. The interaction effect between emotion and identity consistency was not significant, *F*(1, 47) = 1.599, *p* = 0.212, and $\eta_{p}^{2}$ = 0.033. The interaction effect between emotion and estimation problem was not significant, *F*(1, 47) = 0.010, *p* = 0.919, and $\eta_{p}^{2}$ = 0.000. The three-way interaction effect was not significant, *F*(1, 47) = 0.460, *p* = 0.501, and $\eta_{p}^{2}$ = 0.010. The specific numerical values of mean proportions for completing estimation tasks under different conditions are shown in Table 3.

For response times, the interaction effect between emotion and identity consistency was significant, *F*(1, 47) = 10.418, *p* = 0.002, and $\eta_{p}^{2}$ = 0.181. The simple effect analysis showed that, under identity consistency conditions, response times were longer in the happy condition than in the fear condition (*p* = 0.002) and longer in the neutral condition than in the fear condition (*p* < 0.001). Under the happy condition, response times was shorter under identity consistency than under identity inconsistency (*p* = 0.003). Under the fear condition, response times was shorter under identity consistency than under identity inconsistency (*p* < 0.001). The interaction effect between estimation problem and identity consistency was significant, *F*(1, 47) = 4.189, *p* = 0.046, and $\eta_{p}^{2}$ = 0.082. The simple effect analysis showed that for UD problems, response times were shorter under identity consistency than under identity inconsistency (*p* < 0.001). Under identity consistency conditions, response times for UD problems were shorter than for DU problems (*p* = 0.038). The main effect of emotion was significant, *F*(1, 47) = 5.078, *p* = 0.029, and $\eta_{p}^{2}$ = 0.098. The main effect of identity consistency was significant, F(1, 47) = 19.625, *p* = 0.000, and $\eta_{p}^{2}$ = 0.295. The main effect of estimation problem was not significant, *F*(1, 47) = 0.813, *p* = 0.372, and $\eta_{p}^{2}$ = 0.017. The interaction effect between emotion and estimation problem was not significant, *F*(1, 47) = 0.248, *p* = 0.621, and $\eta_{p}^{2}$ = 0.005. The three-way interaction effect was not significant, *F*(1, 47) = 0.756, *p* = 0.389, and $\eta_{p}^{2}$ = 0.016. The specific numerical values of response times for completing estimation tasks under different conditions are shown in Table 3. Additionally, the Pearson correlation analysis revealed no significant correlation between mean proportions and response times under different conditions (*ps* > 0.05) (Table 3), indicating that this study found no trade-off between response times and mean proportions.

#### 3.2.2. The Moderating Role of Math Anxiety in the Influence of Emotions on Estimation Performance

The results of Pearson correlation analysis showed that there was no significant correlation between mean estimation proportions and estimation response times and mathematics anxiety (*ps* > 0.05) (Table 4), indicating that mathematical anxiety generally did not influence the estimation performance of the participants.

In summary, Experiment 2 demonstrated that, first, individuals’ mean estimation proportions was influenced by identity consistency and estimation strategy, while the corresponding response times were not only affected by identity consistency and estimation strategy but also by the interaction between emotion and identity consistency. Second, there were no correlations between individuals’ performance in estimation tasks and their level of math anxiety under different experimental conditions. Based on the known influence of facial emotion consistency and identity consistency on estimation problems, we further explored whether there were interactions between facial emotion consistency and identity consistency. To this end, we designed relevant experiments for verification.

## 4. Experiment 3: The Influence of Dual Information Processing of Facial Emotion and Identity on Estimation Performance

### 4.1. Method

#### 4.1.1. Participants

The method for calculating the sample size in Experiment 3 was the same as in Experiment 1. A total of 52 college students between 18 and 24 (M ± SD, 19.82 ± 1.12) years of age were recruited, with 2 participants excluded due to equipment malfunction and file loss, leaving 50 subjects. These participants were both right-handed with normal or corrected-to-normal vision. This study was conducted according to the ethical principles of the Declaration of Helsinki and received approval from the Research Ethics Committee of the School of Education Science of Yangzhou University (JKY-2023032701). Following a detailed explanation of this study’s procedures, written consent was obtained from each participant. In return for their involvement, participants were provided with a small amount of money.

#### 4.1.2. Materials

The materials used in Experiment 3 were the same as those used in Experiment 1. When the emotions expressed by the faces were consistent, there were three scenarios: happy–happy, neutral–neutral, and fearful–fearful. These were further divided into two categories based on identity consistency (consistent identity and inconsistent identity), meaning there were six scenarios in total. When the emotions expressed by the faces were inconsistent, there were six scenarios: happy–neutral, happy–fearful, neutral–fearful, neutral–happy, fearful–happy, and fearful–neutral. These were again divided into two categories based on identity consistency (consistent identity and inconsistent identity), resulting in another 12 scenarios. Therefore, there were a total of 18 types, with 12 images for each type.

#### 4.1.3. Procedure

**Figure 3:**
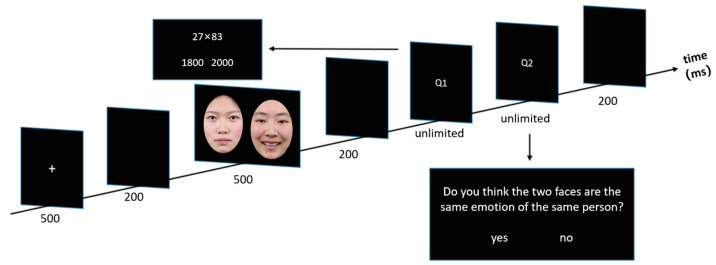
Trial structure in Experiment 3. The facial expression images used in the actual experiment were sourced from the NimStim Facial Expression Set, but due to copyright restrictions, the images in the figure are provided by the authors (Experiment 3).

The experimental procedure was the same as in Experiment 1, except that the emotional consistency judgment task in Experiment 1 was replaced by an emotion and identity consistency judgment (EIJ) task. In the EIJ task, if the two faces displayed the same individual with the same emotion (happy–happy, neutral–neutral, and fearful–fearful), participants were required to press the “F” key with their left middle finger. If the faces showed the same individual with different emotions, different individuals with the same emotion, or different individuals with different emotions, they were required to press the “J” key with their right index finger. Figure 3 illustrates the order in which stimuli were presented during a single trial.

#### 4.1.4. Data Analysis

The data analysis method used in Experiment 3 was the same as that used in Experiment 1.

### 4.2. Results

#### 4.2.1. Mean Proportions and Response Times When Completing the MCE Task

Following the pattern established in Experiment 1, we analyzed mean proportions and response times using a 3 (emotion types: happy, neutral, and fearful) × 2 (emotional consistency: emotional consistent and emotional inconsistent) × 2 (identity consistency: identity consistent and identity inconsistent) × 2 (estimation problem types: DU and UD) experimental design repeated-measure ANOVA. The results are detailed below.

For mean proportions, the interaction effect between emotion and emotional consistency was significant, *F*(1, 49) = 6.707, *p* = 0.013, $\eta_{p}^{2}$ = 0.120. The simple effect analysis showed that, under emotional inconsistency conditions, mean proportions were higher in the happy condition than in the neutral condition (*p* = 0.002), higher in the happy condition than in the fear condition (*p* = 0.029), and lower in the neutral condition than in the fear condition (*p* = 0.038). Under the happy condition, mean proportions were lower under emotional consistency than under emotional inconsistency (*p* < 0.001). Under the neutral condition, mean proportions were lower under emotional consistency than under emotional inconsistency (*p* < 0.001). Under the fearful condition, mean proportions was lower under emotional consistency than under emotional inconsistency (*p* < 0.001). The interaction effect between emotion and identity consistency was significant, *F*(1, 49) = 16.071, *p* < 0.001, $\eta_{p}^{2}$ = 0.247. Simple effects analysis showed that under identity consistency conditions, mean proportions were higher in the happy condition than in the neutral condition (*p* < 0.001), and lower in the neutral condition than in the fear condition (*p* = 0.018). Under identity inconsistency conditions, mean proportions were lower in the happy condition than in the neutral condition (*p* < 0.001), and lower in the happy condition than in the fear condition (*p* = 0.001). Under the happy condition, mean proportions were higher under identity consistency than under identity inconsistency (*p* = 0.004). The interaction effect between emotional consistency and identity consistency was significant, *F*(1, 49) = 6.707, *p* = 0.013, $\eta_{p}^{2}$ = 0.120. Simple effects analysis showed that under identity consistency conditions, mean proportions were lower under emotional consistency than under emotional inconsistency (*p* < 0.001). Under identity inconsistency conditions, mean proportions were lower under emotional consistency than under emotional inconsistency (*p* < 0.001). Under emotional consistency conditions, mean proportions were higher under identity consistency than under identity inconsistency (*p* = 0.030). The main effect of emotion was not significant, *F*(1, 49) = 1.136, *p* = 0.292, $\eta_{p}^{2}$ = 0.023. The main effect of emotional consistency was significant, *F*(1, 49) = 50.901, *p* < 0.001, $\eta_{p}^{2}$ = 0.510. The main effect of identity consistency was not significant, *F*(1, 49) = 3.378, *p* = 0.072, $\eta_{p}^{2}$ = 0.064. The main effect of estimation problem was not significant, *F*(1, 49) = 3.546, *p* = 0.066, $\eta_{p}^{2}$ = 0.067. The interaction effect between emotion and estimation problem was not significant, *F*(1, 49) = 0.065, *p* = 0.799, $\eta_{p}^{2}$ = 0.001. The interaction effect between emotional consistency and estimation problem was not significant, *F*(1, 49) = 0.038, *p* = 0.846, $\eta_{p}^{2}$ = 0.001. The interaction effect between identity consistency and estimation problem was not significant, *F*(1, 49) = 3.984, *p* = 0.052, $\eta_{p}^{2}$ = 0.075. The three-way interaction effects among emotion, emotional consistency, and identity consistency were not significant, *F*(1, 49) = 3.672, *p* = 0.061, $\eta_{p}^{2}$ = 0.07. The three-way interaction effects among emotion, emotional consistency, and estimation problem were not significant, *F*(1, 49) = 0.315, *p* = 0.577, $\eta_{p}^{2}$ = 0.006. The three-way interaction effects among emotion, identity consistency, and estimation problem were not significant, *F*(1, 49) = 0.478, *p* = 0.492, $\eta_{p}^{2}$ = 0.010. The three-way interaction effects among emotional consistency, identity consistency, and estimation problem were not significant, *F*(1, 49) = 0.197, *p* = 0.659, $\eta_{p}^{2}$ = 0.004. The four-way interaction effect was not significant, *F*(1, 49) = 0.485, *p* = 0.490, $\eta_{p}^{2}$ = 0.010. The specific numerical values of mean proportions for completing estimation tasks under different conditions is shown in Table 5.

In terms of response times, the interaction effect between emotional consistency and identity consistency was significant, *F*(1, 49) = 8.236, *p* = 0.006, $\eta_{p}^{2}$ = 0.144. The simple effect analysis showed that, under emotional consistency conditions, response times were shorterfor identity consistency than for identity inconsistency (*p* = 0.003). The interaction effect between emotional consistency and estimation problem was significant, *F*(1, 49) = 5.645, *p* = 0.021, $\eta_{p}^{2}$ = 0.103. The simple effect analysis showed that, for UD problems, response times were longer under emotional consistency than under emotional inconsistency (*p* = 0.040). The main effect of emotion was not significant, *F*(1, 49) = 0.425, *p* = 0.518, $\eta_{p}^{2}$ = 0.009. The main effect of emotional consistency was not significant, *F*(1, 49) = 0.051, *p* = 0.822, $\eta_{p}^{2}$ = 0.001. The main effect of identity consistency was significant, *F*(1, 49) = 5.126, *p* = 0.028, $\eta_{p}^{2}$ = 0.095. The main effect of estimation problem was not significant, *F*(1, 49) = 0.576, *p* = 0.452, $\eta_{p}^{2}$ = 0.012. The interaction effect between emotion and emotional consistency was not significant, *F*(1, 49) = 0.307, *p* = 0.582, $\eta_{p}^{2}$ = 0.006. The interaction effect between emotion and identity consistency was not significant, *F*(1, 49) = 0.565, *p* = 0.456, $\eta_{p}^{2}$ = 0.011. The interaction effect between emotion and estimation problem was not significant, *F*(1, 49) = 0.548, *p* = 0.463, $\eta_{p}^{2}$ = 0.011. The interaction effect between identity consistency and estimation problem was not significant, *F*(1, 49) = 0.045, *p* = 0.833, $\eta_{p}^{2}$ = 0.001. The three-way interaction effects among emotion, emotional consistency, and identity consistency were not significant, *F*(1, 49) = 1.212, *p* = 0.276, $\eta_{p}^{2}$ = 0.024. The three-way interaction effects among emotion, emotional consistency, and estimation problem were not significant, *F*(1, 49) = 0.575, *p* = 0.452, $\eta_{p}^{2}$ = 0.012. The three-way interaction effects among emotion, identity consistency, and estimation problem were not significant, *F*(1, 49) = 1.005, *p* = 0.321, $\eta_{p}^{2}$ = 0.020. The three-way interaction effects among emotional consistency, identity consistency, and estimation problem were not significant, *F*(1, 49) = 0.076, *p* = 0.784, $\eta_{p}^{2}$ = 0.002. The four-way interaction effect was not significant, *F*(1, 49) = 0.985, *p* = 0.326, $\eta_{p}^{2}$ = 0.020. The specific numerical values of response times for completing estimation tasks under different conditions is shown in Table 5. Additionally, the Pearson correlation analysis revealed no significant correlation between mean proportions and response times under different conditions (*ps* > 0.05) (Table 5), indicating that this study found no trade-off between response times and mean proportions.

#### 4.2.2. The Moderating Role of Math Anxiety in the Influence of Emotions on Estimation Performance

The results of the Pearson correlation analysis indicated partial correlations between estimation performance under different emotional conditions and math anxiety. Specifically, when participants faced happy faces with emotional and identity consistency, mean estimation proportions for DU problems were positively correlated with math anxiety (*p* = 0.049). When participants faced fearful faces with emotional consistency but identity inconsistency, mean estimation proportions for DU problems were negatively correlated with math anxiety (*p* = 0.007). When participants faced happy faces with emotional inconsistency but identity consistency, mean estimation proportions for DU problems were negatively correlated with math anxiety (*p* = 0.048). When participants faced happy faces with both emotional and identity inconsistency, mean estimation proportions for DU problems were negatively correlated with math anxiety (*p* = 0.010). When participants faced fearful faces with emotional inconsistency but identity consistency, mean estimation proportions for DU problems were negatively correlated with math anxiety (*p* = 0.046). When participants faced fearful faces with both emotional and identity inconsistency, mean estimation proportions for DU problems were negatively correlated with math anxiety (*p* = 0.005). When participants faced fearful faces with emotional and identity consistency, estimation response times for UD problems were negatively correlated with math anxiety (*p* = 0.011) (Table 6).

In summary, Experiment 3 demonstrated that, first, individuals’ mean estimation proportions and response times were more readily influenced by the interaction between emotional consistency and identity consistency. Second, under different experimental conditions, there were partial correlations between individuals’ performance in estimation tasks and their level of math anxiety, which were mainly manifested as negative correlations between mean proportions and math anxiety under happy and fearful conditions.

## 5. Discussion

The objective of this research was to explore the effects of facial information processing in emotion and identity fields in estimation performance. Concurrently, the relationship between mathematics anxiety and the estimation performance was also examined to confirm whether individuals’ selections of estimation answers were influenced by mathematics anxiety. In Experiments 1 and 2, the effects of facial emotional and identity information processing on participants’ efficiency in applying estimation problems were observed during the completion of the MCE task. Subsequently, in Experiment 3, the effects of the interaction between facial emotional information and identity information on their efficiency in applying estimation problems were observed throughout the MCE task. In the three experiments, individuals’ mean estimation proportions and response times were more easily influenced by the consistency of facial information rather than the type of estimation problems. At the same time, individuals’ math anxiety moderated the influence of emotions on estimation performance.

### 5.1. The Influence of Facial Information in Estimation Performance

In Experiment 1, we found that individuals’ mean estimation proportions and response times were more readily influenced by the interaction between emotion and emotional consistency. Specifically, mean proportions were higher under the happy condition than under the neutral condition, and response times were shorter under the happy condition than under the neutral condition. This indicates that the estimation efficiency was higher when participants faced happy faces compared to neutral faces. Similarly, estimation efficiency was higher when participants faced happy faces than when they faced fearful faces. This supports Hypothesis 1, which states that negative emotions impair estimation performance, while positive emotional experiences enhance it (Fabre & Lemaire, 2019; Liu et al., 2021). Additionally, under different emotional conditions, response times were shorter for emotionally congruent faces than for emotionally incongruent faces. This suggests that estimation efficiency was higher when participants faced emotionally congruent faces than when they faced emotionally incongruent faces. This aligns with the prediction of Hypothesis 2 and confirms the emotional congruence effect (Stenberg et al., 1998).

In Experiment 2, individuals’ mean estimation proportions were influenced by the interaction between identity consistency and estimation strategy, while the corresponding response times were affected not only by the interaction between identity consistency and estimation strategy but also by the interaction between emotion and identity consistency. Specifically, under identity consistency conditions, mean proportions were lower for UD problems than for DU problems, and response times were shorter for UD problems than for DU problems. This indicates that the impact of identity consistency and estimation problems on estimation performance is complex. When identity was consistent, response times were longer under the happy condition than under the fear condition, suggesting that estimation efficiency was lower when participants faced happy faces compared to fearful faces. Similarly, estimation efficiency was lower when participants faced neutral faces than when they faced fearful faces, indicating that negative emotions can enhance estimation efficiency when identity is consistent, which is contrary to Hypothesis 1. Additionally, under happy and fearful conditions, response times were shorter under identity consistency than under identity inconsistency, suggesting that estimation efficiency was higher when participants faced identity-consistent faces than when they faced identity-inconsistent faces. This is inconsistent with Hypothesis 3, as identity consistency not only affects response times but is also influenced by emotion. This finding is inconsistent with the study by Ganel and Goshen-Gottstein (2004), who argued that significant facial emotions do not affect facial identity judgments, but facial identity does affect facial emotion judgments. This suggests a complex interplay between facial emotion and identity consistency.

In Experiment 3, we found that individuals’ mean estimation proportions were influenced by the interactions between emotion and emotional consistency, emotion and identity consistency, and emotional consistency and identity consistency. The corresponding response times were more readily affected by the factors of emotional consistency and identity consistency. Specifically, when emotional inconsistency was present, mean proportions were higher under the happy condition than under the neutral condition, indicating that estimation efficiency was higher when participants faced happy faces compared to neutral faces. Similarly, estimation efficiency was higher when participants faced happy faces than when they faced fearful faces, but lower when they faced neutral faces compared to fearful faces. This contradicts Hypothesis 1, suggesting that the impact of emotion on estimation efficiency is complex. Under identity consistency conditions, mean proportions were higher under the happy condition than under the neutral condition, indicating that estimation efficiency was higher when participants faced happy faces compared to neutral faces. Similarly, estimation efficiency was higher when participants faced fearful faces compared to neutral faces. Under identity inconsistency conditions, estimation efficiency was higher when participants faced neutral faces compared to happy faces, and higher when they faced fearful faces compared to happy faces. Under the happy condition, mean proportions were higher under identity consistency than under identity inconsistency, indicating that estimation efficiency was higher when participants faced identity-consistent faces compared to identity-inconsistent faces. Under different identity consistency conditions, mean proportions were lower for emotionally congruent faces than for emotionally incongruent faces, suggesting that estimation efficiency was lower when participants faced emotionally congruent faces compared to emotionally incongruent faces. This is contrary to Hypothesis 2. Under emotional consistency conditions, mean proportions were higher under identity consistency than under identity inconsistency, and response times were shorter under identity consistency than under identity inconsistency, indicating that estimation efficiency was higher when participants faced identity-consistent faces compared to identity-inconsistent faces. This also contradicts Hypothesis 3, as emotional consistency affects response times under identity consistency, and additionally, mean emotional consistency proportions are influenced by identity consistency, mean identity consistency proportions are influenced by emotional consistency, mean emotional proportions are influenced by identity consistency, and mean identity consistency proportions are influenced by emotion.

### 5.2. The Moderating Role of Math Anxiety in the Influence of Emotions on Estimation Performance

Experiment 1 indicated that, under different experimental conditions, there were partial correlations between individuals’ performance in estimation tasks and their level of math anxiety, which were mainly manifested as negative correlations between mean proportions and math anxiety under happy and fearful conditions when emotional inconsistency was present. Experiment 2 showed that, under different experimental conditions, there were no correlations between individuals’ performance in estimation tasks and their level of math anxiety. Experiment 3 demonstrated that, under different experimental conditions, there were partial correlations between individuals’ performance in estimation tasks and their level of math anxiety, which were mainly manifested as negative correlations between mean proportions and math anxiety under happy and fearful conditions.

Overall, the three experiments suggest that, in most conditions of face information processing, participants’ estimation performance is not affected by their level of math anxiety. However, in a small number of cases, there is a negative correlation between mean proportions under happy and fearful conditions and math anxiety. This finding is inconsistent with Hypothesis 4 and also contradicts the previous research conclusions that math anxiety has no significant impact on the mean proportions and response times of estimation problems (Si et al., 2014). Additionally, it supplements the understanding that math anxiety plays a moderating role in the influence of happy and fearful emotions on mean estimation proportions.

The lack of significant impact of math anxiety on estimation performance may be due to several reasons. For instance, the measurement tools for math anxiety might not be sensitive enough, or the sample we tested was highly selective, with insufficient variation in math anxiety levels. Moreover, although this study found a negative correlation between mean proportions under happy and fearful conditions and math anxiety, the research by Si et al. (2014) discovered that individuals with high levels of math anxiety exhibited larger N100 and N400 amplitudes when completing estimation answer selection tasks. This suggests that math anxiety may influence the selection of estimation answers at the neurophysiological level, and this impact may not be directly reflected in behavioral indicators. Therefore, future research could employ electrophysiological techniques such as ERP and fMRI to more deeply explore the relationship between math anxiety and estimation answer selection. Additionally, a significant limitation of this study regarding the measurement of math anxiety is the lack of an independent arithmetic test (e.g., French Kit, TTR, or other paper-and-pencil tests) to assess arithmetic fluency, to ensure that low-anxiety and high-anxiety individuals differ only in anxiety levels, while other conditions remain the same (including mathematical fluency). Otherwise, we cannot determine whether participants’ math anxiety is unrelated to arithmetic fluency. We will avoid such shortcomings in future research.

Finally, although this study explored the relationship between facial emotional consistency and identity consistency, the exact nature and specific mechanisms of this relationship remain unclear, such as how they interact at the physiological level. This suggests that future research needs to employ more diverse methods, such as ERP and fMRI, to comprehensively understand how math anxiety affects estimation performance.

## 6. Conclusions

The present study found that face information processing (including emotion, emotional consistency, and identity consistency) has a significant impact on individuals’ estimation performance. Moreover, in most conditions of face information processing, participants’ estimation performance is not affected by their level of math anxiety. However, in a small number of cases, there is a negative correlation between mean proportions under happy and fearful conditions and math anxiety.

## Figures and Tables

**Table 1:** Mean proportions and response times when completing the MCE task under different conditions with a single ANOVA including “consistency” variable. The Pearson correlations between mean proportions and response times under different conditions (Experiment 1).

			Happy	Neutral	Fearful
Mean proportions	UD	Emotional consistent	0.912	0.820	0.783
		Emotional inconsistent	0.886	0.745	0.766
	DU	Emotional consistent	0.913	0.862	0.797
		Emotional inconsistent	0.910	0.805	0.803
Reaction time	UD	Emotional consistent	3099	3177	3546
		Emotional inconsistent	3439	3638	3519
	DU	Emotional consistent	2955	3289	3358
		Emotional inconsistent	3486	3558	3639
The Pearson correlations	UD	Emotional consistent	−0.130 (0.374)		
		Emotional inconsistent	0.043 (0.771)		
	DU	Emotional consistent	−0.148 (0.310)		
		Emotional inconsistent	−0.085 (0.562)		
F	Mean correct products	Consistent problems	0.358		
		Inconsistent problems	1.483		
	Mean best estimates	Consistent problems	0.322		
		Inconsistent problems	1.456		

Note: In the same cell of the Pearson correlations, the first number refers to the correlation coefficient, while the second number refers to the *p*-value. All correlation coefficients were non-significant. The same results came out when correlations are calculated separately for each condition.

**Table 2:** The Pearson correlations between estimation performance and mathematics anxiety (Experiment 1).

		Happy Emotional Consistent	Fearful Emotional Consistent	Happy Emotional Inconsistent	Fearful Emotional Inconsistent
Mean proportions	UD	−0.145 (0.321)	0.017 (0.909)	−0.366 (0.010) **	−0.322 (0.024) *
	DU	−0.277 (0.054)	−0.026 (0.861)	−0.370 (0.009) **	−0.266 (0.065)
Reaction time	UD	0.117 (0.424)	0.001 (0.996)	0.021 (0.885)	0.031 (0.832)
	DU	0.255 (0.078)	0.059 (0.687)	0.116 (0.429)	−0.073 (0.619)

Note: In the same cell, the first number refers to the correlation coefficient, while the second number refers to the *p*-value. “*” means “*p* < 0.05”, “**” means “*p* < 0.01”.

**Table 3:** Mean proportions and response times when completing the MCE task under different conditions with a single ANOVA including “consistency” variable. The Pearson correlations between mean proportions and response times under different conditions (Experiment 2).

			Happy	Neutral	Fearful
Mean proportions	UD	Identity consistent	0.867	0.862	0.873
		Identity inconsistent	0.880	0.914	0.895
	DU	Identity consistent	0.895	0.910	0.899
		Identity inconsistent	0.898	0.907	0.897
Reaction time	UD	Identity consistent	2840	2846	2718
		Identity inconsistent	3059	2919	2981
	DU	Identity consistent	2896	2985	2766
		Identity inconsistent	2958	2962	2969
The Pearson correlations	UD	Identity consistent	0.100 (0.500)		
		Identity inconsistent	−0.098 (0.509)		
	DU	Identity consistent	−0.066 (0.657)		
		Identity inconsistent	−0.177 (0.229)		
F	Mean correct products	Consistent problems	0.356		
		Inconsistent problems	0.364		
	Mean best estimates	Consistent problems	0.315		
		Inconsistent problems	0.344		

Note: In the same cell of the Pearson correlations, the first number refers to the correlation coefficient, while the second number refers to the *p*-value. All correlation coefficients were non-significant. The same results came out when correlations are calculated separately for each condition.

**Table 4:** The Pearson correlations between estimation performance and mathematics anxiety (Experiment 2).

		Happy Identity Consistent	Fearful Identity Consistent	Happy Identity Inconsistent	Fearful Identity Inconsistent
Mean proportions	UD	0.020 (0.894)	−0.074 (0.617)	0.074 (0.616)	0.059 (0.689)
	DU	−0.098 (0.506)	−0.201 (0.171)	0.246 (0.092)	0.208 (0.155)
Reaction time	UD	0.070 (0.635)	0.116 (0.431)	0.014 (0.925)	0.048 (0.748)
	DU	−0.105 (0.476)	−0.06 (0.687)	−0.081 (0.582)	−0.163 (0.267)

Note: In the same cell, the first number refers to the correlation coefficient, while the second number refers to the *p*-value. All correlation coefficients were non-significant.

**Table 5:** Mean proportions and response times when completing the MCE task under different conditions with a single ANOVA including “consistency” variable. The Pearson correlations between mean proportions and response times under different conditions (Experiment 3).

				Happy	Neutral	Fearful
Mean proportions	UD	Emotional consistent	Identity consistent	0.904	0.893	0.898
			Identity inconsistent	0.744	0.820	0.787
		Emotional inconsistent	Identity consistent	0.963	0.939	0.944
			Identity inconsistent	0.954	0.946	0.957
	DU	Emotional consistent	Identity consistent	0.900	0.827	0.889
			Identity inconsistent	0.738	0.811	0.804
		Emotional inconsistent	Identity consistent	0.943	0.921	0.922
			Identity inconsistent	0.955	0.944	0.952
Reaction time	UD	Emotional consistent	Identity consistent	2952	2900	2929
			Identity inconsistent	2970	3026	3444
		Emotional inconsistent	Identity consistent	2892	2987	2938
			Identity inconsistent	2870	2909	3001
	DU	Emotional consistent	Identity consistent	2883	2689	2812
			Identity inconsistent	3084	2976	2991
		Emotional inconsistent	Identity consistent	2948	3072	2903
			Identity inconsistent	3008	3059	2938
The Pearson correlations	UD	Emotional consistent	Identity consistent	0.120 (0.408)		
			Identity inconsistent	−0.197 (0.171)		
		Emotional inconsistent	Identity consistent	−0.463 (0.001)		
			Identity inconsistent	−0.290 (0.041)		
	DU	Emotional consistent	Identity consistent	−0.122 (0.399)		
			Identity inconsistent	−0.207 (0.148)		
		Emotional inconsistent	Identity consistent	0.049 (0.733)		
			Identity inconsistent	−0.301 (0.033)		
F	Mean correct products		Consistent problems	0.042		
			Inconsistent problems	0.483		
	Mean best estimates		Consistent problems	0.060		
			Inconsistent problems	0.453		

Note: In the same cell of the Pearson correlations, the first number refers to the correlation coefficient, while the second number refers to the *p*-value. All correlation coefficients were non-significant. The same results came out when correlations are calculated separately for each condition.

**Table 6:** The Pearson correlations between estimation performance and mathematics anxiety (Experiment 3).

			Happy Emotional Consistent	Fearful Emotional Consistent	Happy Emotional Inconsistent	Fearful Emotional Inconsistent
Mean proportions	UD	Identity consistent	−0.134 (0.352)	−0.053 (0.714)	−0.104 (0.470)	−0.197 (0.171)
		Identity inconsistent	−0.161 (0.265)	−0.278 (0.050)	−0.190 (0.186)	−0.116 (0.421)
	DU	Identity consistent	0.280 (0.049) *	−0.145 (0.316)	−0.281 (0.048) *	−0.283 (0.046) *
		Identity inconsistent	−0.279 (0.050)	−0.376 (0.007) **	−0.361 (0.010) **	−0.391 (0.005) **
Reaction time	UD	Identity consistent	−0.211 (0.141)	−0.356 (0.011) *	−0.005 (0.973)	−0.167 (0.248)
		Identity inconsistent	0.134 (0.355)	0.111 (0.443)	−0.066 (0.651)	−0.117 (0.420)
	DU	Identity consistent	0.022 (0.881)	0.087 (0.548)	0.181 (0.208)	−0.065 (0.655)
		Identity inconsistent	0.104 (0.470)	0.180 (0.211)	0.099 (0.492)	0.208 (0.146)

Note: In the same cell, the first number refers to the correlation coefficient, while the second number refers to the *p*-value. “*” means “*p* < 0.05”, “**” means “*p* < 0.01”.

## Data Availability

The datasets used and/or analyzed during the current study are available from the corresponding author (psyclzhu@yzu.edu.cn) upon reasonable request.
